# Supplementary material for: Serum Proteomic Analysis for New Types of Long-Term Persistent COVID-19 Patients in Wuhan
Source: Microbiol Spectr. 2022 Oct 31;10(6):e01270-22. doi: 10.1128/spectrum.01270-22 (PMC9784772; doi:10.1128/spectrum.01270-22)
Supplement: Supplemental file 2 — Fig. S1 to S6. Download spectrum.01270-22-s0002.pdf, PDF file, 1.0 MB [file spectrum.01270-22-s0002.pdf]

Supplementary Figures

Serum proteomic analysis for a new type of long-term persistent COVID-19 patients in Wuhan

Cuidan Li[1#], Liya Yue[1#], Yingjiao Ju[1,2#], Jie Wang[1,2], Mengfan Chen[1,2], Hao Lu[1,2], Sitong Liu[1,2], Tao Liu[1,2], Jing Wang[3], Xin Hu[3], Bahetibieke Tuohetaerbaike[3], Hao Wen[3], Wenbao Zhang[3], Sihong Xu[4], Chunlai Jiang[5], Fei Chen[1,2,3,6]*

Figure S1

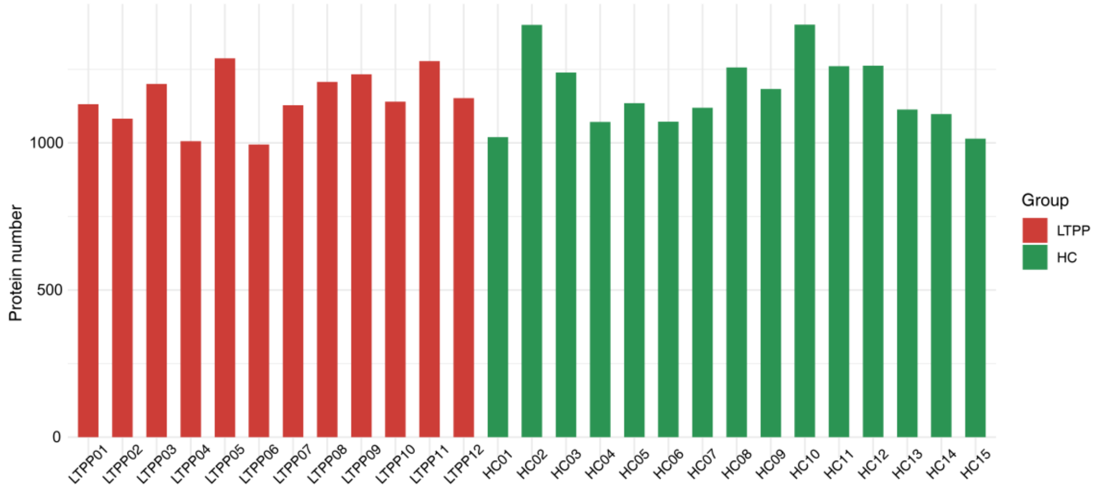

Figure S1 The identified protein numbers in each sample of LTPPs and HCs.

Figure S2

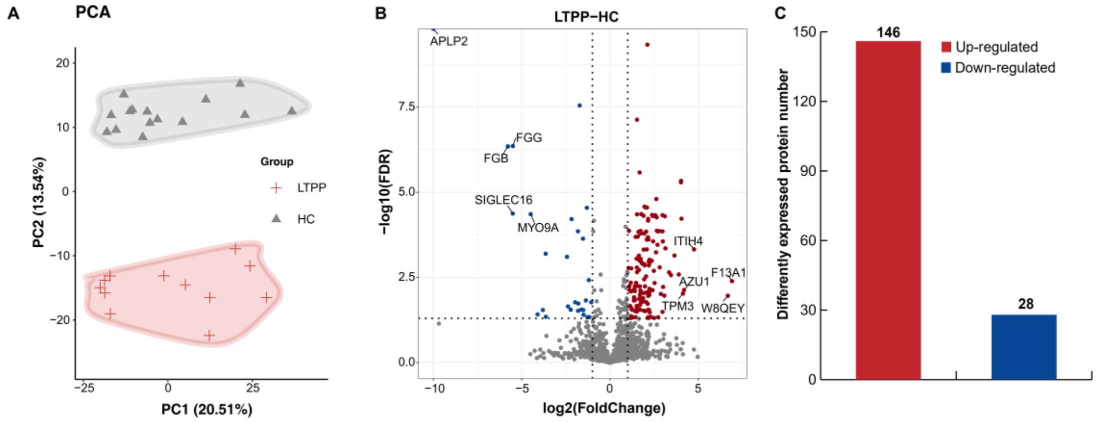

Figure S2 The proteomic profiling of serum from LTPPs and HCs. (A) Principal component analysis of the expressed proteins between the two groups. (B) Volcano plot showing the significantly upregulated (red dots) and downregulated (blue dots) proteins between the two groups (FC > 2). (C) Bar plot representing the number of differently expressed proteins (DEPs).

Figure S3

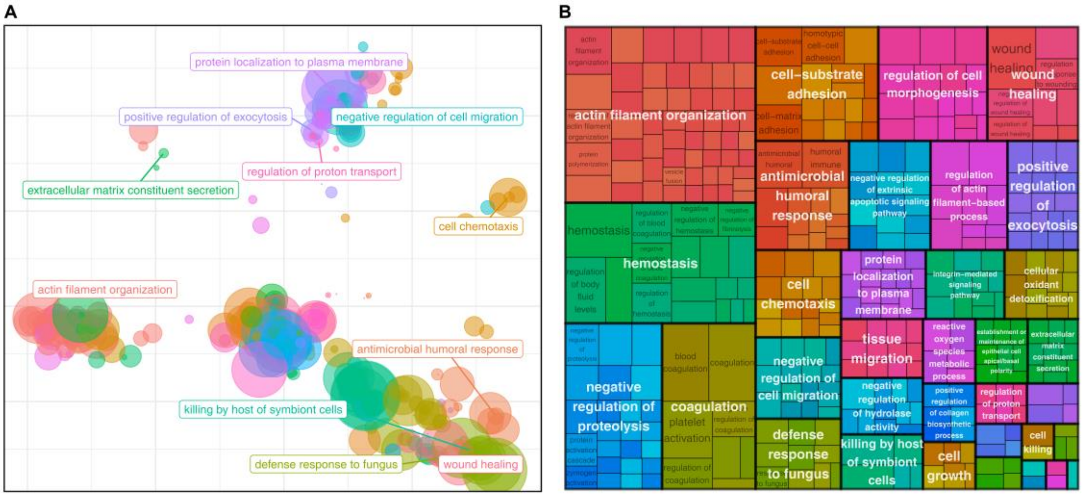

Figure S3 The bubble plot (A) and treemap (B) showing 34 clusters of GO biological processes for the 12 LTPPs.

Figure S4

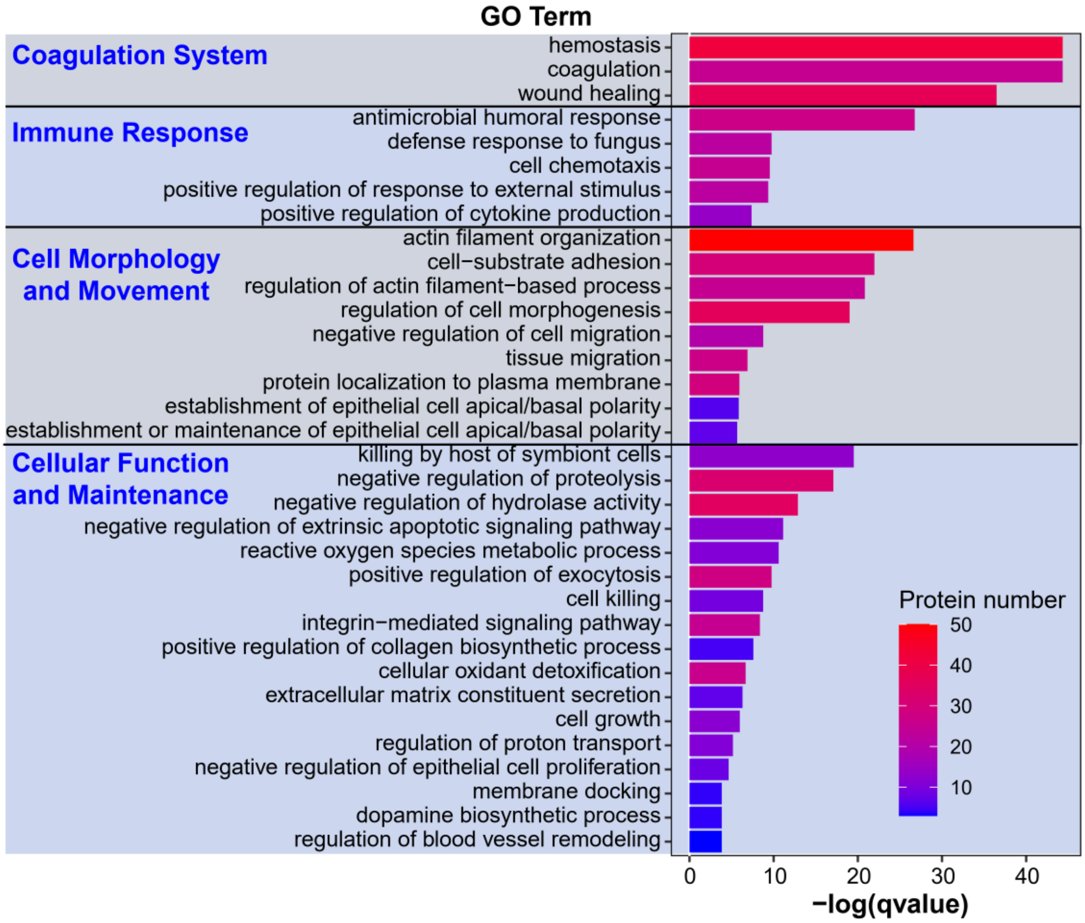

Figure S4 The significantly enriched GO biological processes and the corresponding DEPs of the 12 LTPPs.

Figure S5

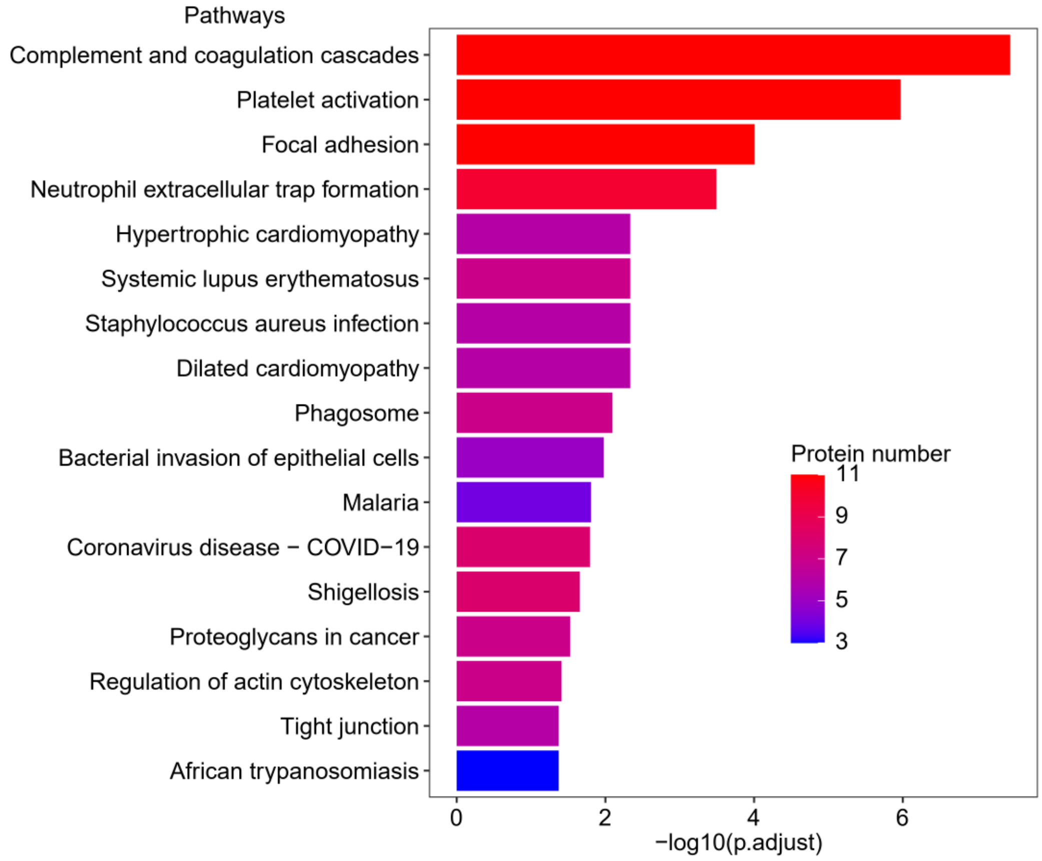

Figure S5 Bar plot showing 17 significantly enriched KEGG pathways for all the DEPs of LTPPs.

Figure S6

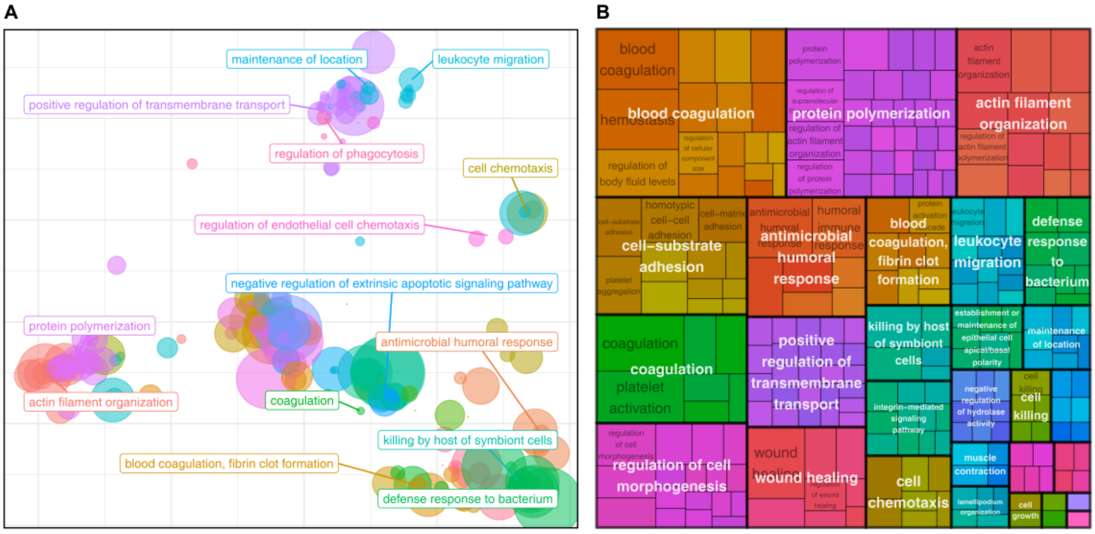

Figure S6 The bubble plot (A) and treemap (B) showing 28 clusters of GO biological processes for the seven LTPPs-NH.
